# Knowledge, trust, and multidimensional perceptions of human papillomavirus vaccination among women attending a single primary care centre in Astana, Kazakhstan: a cross-sectional study

**DOI:** 10.3389/fgwh.2026.1960252

**Published:** 2026-10-08

**Authors:** Ayagyoz Umbetzhanova, Adina Kusniyeva, Gulmira Derbissalina, Aigul Abduldayeva, Nikolay Safonov, Vitaliy Koikov

**Affiliations:** 1 Department of General Medical Practice with a course in evidence-based medicine, Astana Medical University, Astana, Kazakhstan; 2 Department of Research Institute of Preventive Medicine Named Academician E.Dalenov, Astana Medical University, Astana, Kazakhstan; 3 Department of Science, Medical Education and Human Resources, Ministry of Healthcare of the Republic of Kazakhstan, Astana, Kazakhstan

**Keywords:** Central Asia, HPV vaccination, human papillomavirus, Kazakhstan, knowledge, trust, vaccine confidence, vaccine safety

## Abstract

**Background:**

Kazakhstan reintroduced human papillomavirus (HPV) vaccination in 2024, making public confidence and communication important. This study described HPV knowledge and trust in information sources and exploratorily examined vaccine-perception structure and correlates among women attending primary care in Astana.

**Methods:**

A cross-sectional electronic survey was conducted from 17 January to 28 February 2026 among 221 adult women recruited through convenience sampling. HPV knowledge, source-specific trust, and 16 perception items were assessed. Exploratory psychometric analysis used polychoric correlations, parallel analysis, and principal-axis factor analysis with oblimin rotation to derive preliminary composite measures. Spearman correlations and robust multivariable linear models examined safety concern and perceived effectiveness. A secondary logistic model examined refusal or intention to refuse HPV vaccination for a daughter.

**Results:**

Mean HPV knowledge was 4.95 ± 3.84 of 12. A five-item safety concern measure (mean 2.34 ± 0.97; *α* = 0.868; *ω* = 0.901) and a three-item perceived effectiveness measure (mean 3.20 ± 1.24; *α* = 0.922; *ω* = 0.948) were derived. Knowledge correlated negatively with safety concern (*ρ* = −0.284, *p* < 0.001) and positively with perceived effectiveness (*ρ* = 0.393, *p* < 0.001). Safety concern was associated with COVID-19 controversy spillover (B = 0.190, 95% CI 0.043−0.337), earlier-programme distrust (B = 0.197, 95% CI 0.047−0.347), and preference for natural immunity (B = 0.137, 95% CI 0.040−0.234). Higher knowledge (B = 0.061, 95% CI 0.010−0.111) and professional/scientific trust (B = 0.297, 95% CI 0.114−0.480) were associated with greater perceived effectiveness. Forty-nine participants (22.2%) reported refusal or intention to refuse vaccination for a daughter; knowing someone believed to have become seriously ill after vaccination was associated with this response (aOR 7.42, 95% CI 2.60−21.16).

**Conclusion:**

HPV vaccine perceptions were multidimensional. Knowledge and professional-scientific trust were linked mainly to perceived effectiveness, whereas safety concerns were more strongly related to vaccine narratives. Communication strategies should combine factual education with transparent safety dialogue and clinician communication.

## 1 Introduction

Human papillomavirus is a necessary cause of nearly all cervical cancers and also contributes to anal, vulvar, vaginal, penile, and oropharyngeal cancers. In 2022, cervical cancer accounted for approximately 660,000 new cases and 350,000 deaths worldwide, with the largest burden concentrated in low- and middle-income countries (1, 2). Prophylactic HPV vaccines are highly effective when administered before exposure to vaccine-targeted HPV types, and population studies have demonstrated substantial reductions in cervical precancer and invasive cervical cancer after programme implementation (3–5).

The World Health Organization's cervical cancer elimination strategy sets the 2030 target of vaccinating 90% of girls against HPV by age 15, screening 70% of women with a high-performance test, and treating 90% of women with cervical precancer or cancer (2). Within this global prevention framework, Kazakhstan has a distinctive implementation history. An HPV vaccination pilot was introduced in four regions during 2013−2014 but was later discontinued after low uptake and widespread parental refusal in a context of negative media coverage, safety concerns, and communication gaps (15, 16, 21). The national programme was relaunched in September 2024 for 11-year-old girls, with catch-up vaccination for girls aged 12−13 years and parental informed consent (6, 7). In May 2026, additional voluntary HPV vaccination was extended to girls aged 14−17 years inclusive (26). This sequence makes locally grounded evidence on knowledge, trust, safety perceptions, and communication directly relevant to the renewed programme.

Factual knowledge is an important component of HPV vaccine decision-making, but it operates alongside perceived safety and effectiveness, healthcare-provider recommendation, trust, social norms, convenience, and previous vaccine experiences (8–10). These determinants also sit within the broader concept of vaccine hesitancy, in which acceptance may be delayed, uncertain, or context-dependent rather than explained by knowledge alone (10, 18). Provider communication is among the strongest correlates of HPV vaccine initiation, while safety concerns remain a consistent barrier (9, 11). The post-COVID-19 information environment adds further complexity because vaccine controversies, adverse-event narratives, and institutional mistrust may spill over across otherwise unrelated vaccination programmes (12, 20). Consequently, individuals may recognize vaccine benefits while retaining specific safety concerns.

Evidence from Kazakhstan has expanded but remains limited. Earlier surveys found mixed or low levels of HPV knowledge and approximately half of women or mothers expressing positive vaccine attitudes (13, 14). Research among healthcare professionals identified incomplete knowledge and communication barriers despite generally favourable recommendation intentions (15). A more recent study of primary-care providers in Almaty likewise reported important gaps in knowledge and recommendation practice (16). Most available studies were conducted before or around programme reintroduction, and few have examined whether safety concern and perceived effectiveness operate as separable dimensions within the renewed vaccination context.

The present study therefore aimed to: describe HPV knowledge and trust in vaccine information sources among adult women attending a primary healthcare centre in Astana; exploratorily evaluate the psychometric structure of the available HPV vaccine perception items and derive preliminary composite measures for association analyses; identify factors independently associated with safety concern and perceived effectiveness; and explore factors associated with reported refusal or intention to refuse HPV vaccination for a daughter.

These questions have direct health-communication relevance. Provider recommendation and communication are consistently associated with HPV vaccine uptake, and recent evidence indicates that clinician communication training, particularly approaches combining strong recommendations with structured responses to concerns, can improve vaccination uptake (11, 19, 27). In Kazakhstan, locally tailored communication is especially important because the renewed programme operates alongside historical programme distrust and a predominantly digital information environment (15, 16, 25).

## 2 Materials and methods

### 2.1 Study design and setting

This single-centre cross-sectional study was conducted from 17 January to 28 February 2026 at the City Center for Primary Health Care in Astana, Kazakhstan. Reporting was structured in accordance with the Strengthening the Reporting of Observational Studies in Epidemiology (STROBE) recommendations for cross-sectional studies (17). The unit of analysis was an individual survey respondent.

### 2.2 Participants and data collection

Women aged 18 years or older attending the study centre were eligible. Recruitment was conducted through 14 gynecology offices. Participants were recruited through open convenience sampling. QR codes linking to the electronic questionnaire were displayed in the participating offices, and interested women accessed and completed the survey independently.

Because recruitment was open and exposure to the QR-code invitation was not individually registered, the numbers of women who viewed the invitation, opened the questionnaire, or declined participation could not be determined; consequently, a conventional response rate could not be calculated. To reduce duplicate participation, the Google Forms setting limiting submission to one response per Google account was enabled. Participants were required to sign in to submit the questionnaire; however, email addresses and other Google account identifiers were not collected or made available to the research team.

All questionnaire items were configured as mandatory. Google Forms did not permit submission while any item remained unanswered. No questionnaires were excluded at the analytical stage, and the final dataset comprised 221 fully completed questionnaires with no item-level missing data. No *a priori* sample-size calculation was performed because the study was designed as an exploratory cross-sectional survey; all complete submissions received during the predefined recruitment period were included.

### 2.3 Questionnaire

Data were collected using a structured bilingual questionnaire based on the French Survey Questionnaire on the Determinants of HPV Vaccine Hesitancy (18) and on instruments previously adapted and psychometrically evaluated among women and mothers in Kazakhstan (13, 14). The broader questionnaire framework has also been described in a Kazakhstan mixed-methods study protocol (21). The questionnaire developers provided the validated Russian- and Kazakh-language version and granted written permission for its use in the present study. Each item was presented in Kazakh and Russian within the same electronic form, separated by a slash, allowing participants to respond in their preferred language. Daughter-directed items were explicitly worded to apply both to women who had a daughter and to women considering a future daughter. The questionnaire included sociodemographic and health-related characteristics, HPV and HPV-vaccine awareness, prior vaccination experience, trust in health institutions and information sources, information-seeking behaviour, 12 true/false/don't-know HPV knowledge items, six source-specific trust items, contextual vaccination beliefs, and 16 HPV vaccine perception items. Representative questionnaire items and response formats are provided in Supplementary Table S8.

### 2.4 Scoring and recoding

Before score calculation, numeric category codes were mapped to ordered values consistent with the response options shown in the questionnaire. Agreement items were scored from 1 (fully disagree) to 5 (fully agree), and trust items were scored from 1 (absolute distrust) to 5 (full trust). Positively and negatively framed items were oriented according to the direction of the construct being analysed. The complete scoring map is provided in Supplementary Table S1.

### 2.5 Measures and psychometric evaluation

HPV knowledge was scored as the number of correct responses to 12 items (range 0−12); incorrect and ‘don't know’ responses received zero points. Source-specific trust was summarized as professional/scientific trust (healthcare professionals and scientific researchers), institutional trust (pharmaceutical industry and government), and media trust (traditional and alternative media), each ranging from 1 to 5.

The 16 HPV vaccine perception items were evaluated without assuming that they formed one scale. Because the item set covered several related but conceptually distinct constructs, the psychometric analysis was exploratory and was used to characterize dimensional structure rather than to confirm prespecified latent scales. Suitability for factor analysis was assessed using the Kaiser-Meyer-Olkin statistic and Bartlett's test of sphericity. Factor retention was guided by parallel analysis (23) based on 1,000 random permutations. Principal-axis exploratory factor analysis was applied to a polychoric correlation matrix with oblimin rotation because the five-category items were ordinal and correlated factors were expected. Item selection for subsequent analytical composites considered factor loadings, cross-loadings, uniqueness, item-total behaviour, and conceptual coherence, in accordance with recommended scale-development practices (24).

Based on the exploratory factor structure, item performance, and conceptual coherence, two preliminary study-derived composite measures were defined as the primary regression outcomes. Safety concern was calculated as the mean of five items: possible long-term health problems, insufficient study, the vaccine being too new to judge safety, the daughter being too young, and the belief that non-mandatory status reflected danger. The direct statement that HPV vaccines were unsafe was retained as a standalone item because it correlated inversely with the other concern items and had very high uniqueness. Its wording and response direction were retained from the validated bilingual questionnaire, and its source coding was verified against the scoring framework. Perceived effectiveness was calculated as the mean of three items concerning prevention of HPV infection, genital warts, and HPV-related cancer. Higher scores indicated greater concern or greater perceived effectiveness, respectively. These composites were treated as preliminary analytical measures rather than independently validated scales.

### 2.6 Statistical analysis

Data management, descriptive analyses, Cronbach's alpha, Spearman rank correlations, and regression models were performed using IBM SPSS Statistics version 27.0.1 (IBM Corp., Armonk, NY, USA). Polychoric correlation matrices, parallel analysis, McDonald's omega (22), and publication-quality figures were generated using Python 3 through the IBM SPSS Statistics Integration Plug-in for Python. Categorical variables are presented as frequencies and percentages. Continuous and scale variables are summarized using means and standard deviations and, where informative, medians and interquartile ranges.

Internal consistency was evaluated using Cronbach's alpha and McDonald's omega. Item-total correlations and alpha-if-item-deleted diagnostics were inspected. Associations between scale scores were assessed using Spearman rank correlation. The two primary outcomes were analysed using multivariable normal-identity models with heteroskedasticity-consistent robust covariance estimates. The safety concern model included age, education, healthcare-worker status, having children, previous mandatory vaccination, knowledge, the three trust domains, information-search history, personal anecdotal exposure to serious illness after vaccination or while unvaccinated, COVID-19 controversy spillover, distrust linked to the earlier Kazakhstan HPV programme, belief in alternative medicine, and preference for natural immunity. The effectiveness model used the same demographic, knowledge, trust, information, and anecdotal-experience variables but excluded the four proximal contextual-belief variables to reduce construct overlap.

A secondary multivariable logistic regression model examined reported refusal or intention to refuse HPV vaccination for a daughter. The questionnaire item asked whether the respondent had refused or decided not to vaccinate her daughter and explicitly instructed women without a daughter to answer prospectively with reference to a future daughter. Accordingly, the outcome captures a combined daughter-directed refusal response rather than verified historical vaccine refusal or observed uptake, and all 221 participants contributed to its denominator. Information on whether participants had a daughter, daughters’ ages, and programme eligibility was not collected. Results are expressed as adjusted odds ratios (aORs) with 95% confidence intervals. Apparent model discrimination was summarized using the area under the receiver-operating-characteristic curve, and overall prediction error was summarized using the Brier score. Sensitivity analyses repeated the primary models among non-healthcare workers, participants with children, and participants who had heard of the HPV vaccine. Variance inflation factors were inspected; all were below 3. Statistical tests were two-sided, and *p* < 0.05 was considered statistically significant. Because the analyses were cross-sectional and partly exploratory, effect estimates and confidence intervals were emphasized over isolated *p*-values.

### 2.7 Ethical considerations

The study was approved by the Local Ethics Committee of NJSC Astana Medical University (Protocol No. 2, 16 January 2026). All participants provided electronic informed consent before completing the questionnaire. The study was conducted in accordance with the Declaration of Helsinki.

## 3 Results

### 3.1 Participant characteristics

The analytical dataset contained 221 participants. The largest age groups were 18−24 years (26.7%) and 35−44 years (25.3%). Most participants had higher education (73.3%), were married (57.5%), and were officially employed (68.3%). Ninety participants (40.7%) were healthcare workers. Overall, 188 women (85.1%) reported having searched for HPV-vaccine information and 210 (95.0%) had heard of the HPV vaccine. Forty-nine women (22.2%) reported refusal or intention to refuse HPV vaccination for a daughter, and 42 (19.0%) reported refusing vaccination for themselves. Participant characteristics are summarized in Table 1.

**Table 1:** Characteristics of study participants (*N* = 221).

Characteristic	Category	*n*	%
Age group	18–24	59	26.7
	25–34	36	16.3
	35–44	56	25.3
	45–54	24	10.9
	55–64	24	10.9
	≥65	22	10.0
Education	Higher education	162	73.3
	Secondary education	16	7.2
	Secondary specialized	21	9.5
	Postgraduate	22	10.0
Marital status	Divorced	10	4.5
	Unmarried	78	35.3
	Widowed	6	2.7
	Married	127	57.5
Number of children	0	94	42.5
	1	39	17.6
	2	39	17.6
	3	32	14.5
	≥4	17	7.7
Healthcare worker	Yes	90	40.7
Officially employed	Yes	151	68.3
Insured	Yes	181	81.9
Previous mandatory vaccination	Yes	119	53.8
Chronic disease	Yes	63	28.5
Positive attitude to prevention	Yes	192	86.9
Religious vaccine refusal	Yes	11	5.0
Heard of HPV vaccine	Yes	210	95.0
Searched HPV vaccine information	Yes	188	85.1
Daughter vaccination refusal/intention	Yes	49	22.2
Self-reported vaccine refusal	Yes	42	19.0
Knows serious illness after vaccination	Yes	32	14.5
Knows serious illness while unvaccinated	Yes	86	38.9
Previous abnormal cervical/vaginal smear requiring treatment	Yes	24	10.9

### 3.2 HPV knowledge and trust in information sources

The mean HPV knowledge score was 4.95 ± 3.84 of 12 (median 5, interquartile range 1−8), with high internal consistency (Cronbach's *α* = 0.886). Correct-response proportions varied markedly by item. Recognition that HPV infection may remain unnoticed for years was highest (64.7%), whereas only 19.9% correctly recognized that cervical cancer may still occur after HPV vaccination. Knowledge that the vaccine protects against genital warts was correct in 29.9%, and 36.7% correctly stated that vaccinated girls still require cervical screening. ‘Don't know’ responses exceeded 40% for 10 of the 12 items, indicating substantial uncertainty rather than uniformly held false beliefs. The item-level HPV knowledge profile is shown in Figure 1.

**Figure 1:**
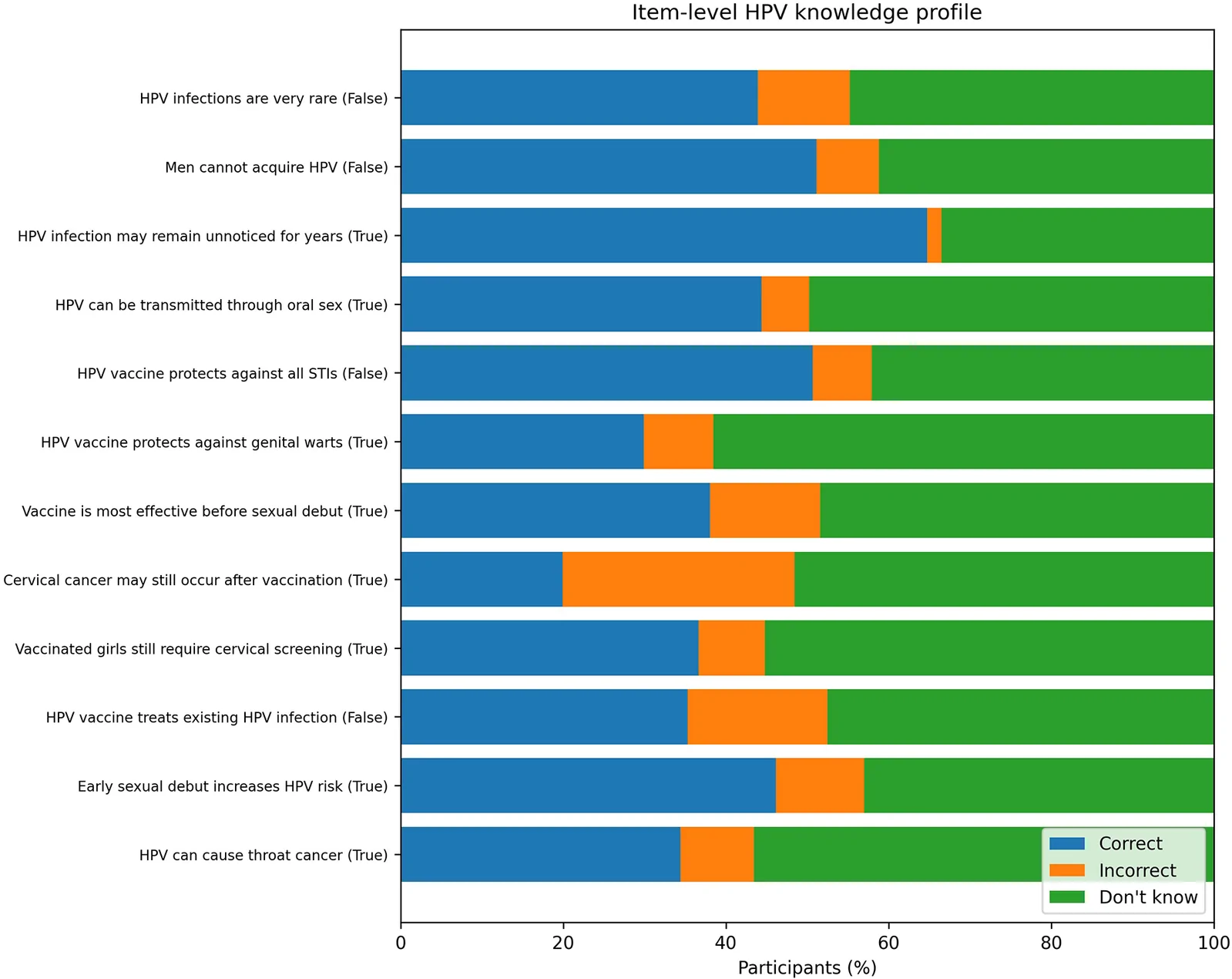
Item-level HPV knowledge profile. Bars show the proportions of correct, incorrect, and ‘don't know’ responses.

Trust differed substantially by source. Mean trust was highest for scientific researchers (3.47 ± 1.40) and healthcare workers (3.29 ± 1.31), while alternative media and social networks had the lowest mean score (2.36 ± 1.05), followed by traditional media (2.48 ± 1.04). The six-item overall trust scale had good internal consistency (*α* = 0.875; *ω* = 0.903). Domain scores were retained because professional/scientific, institutional, and media sources have different communication roles. Mean trust scores by information source are shown in Figure 2.

**Figure 2:**
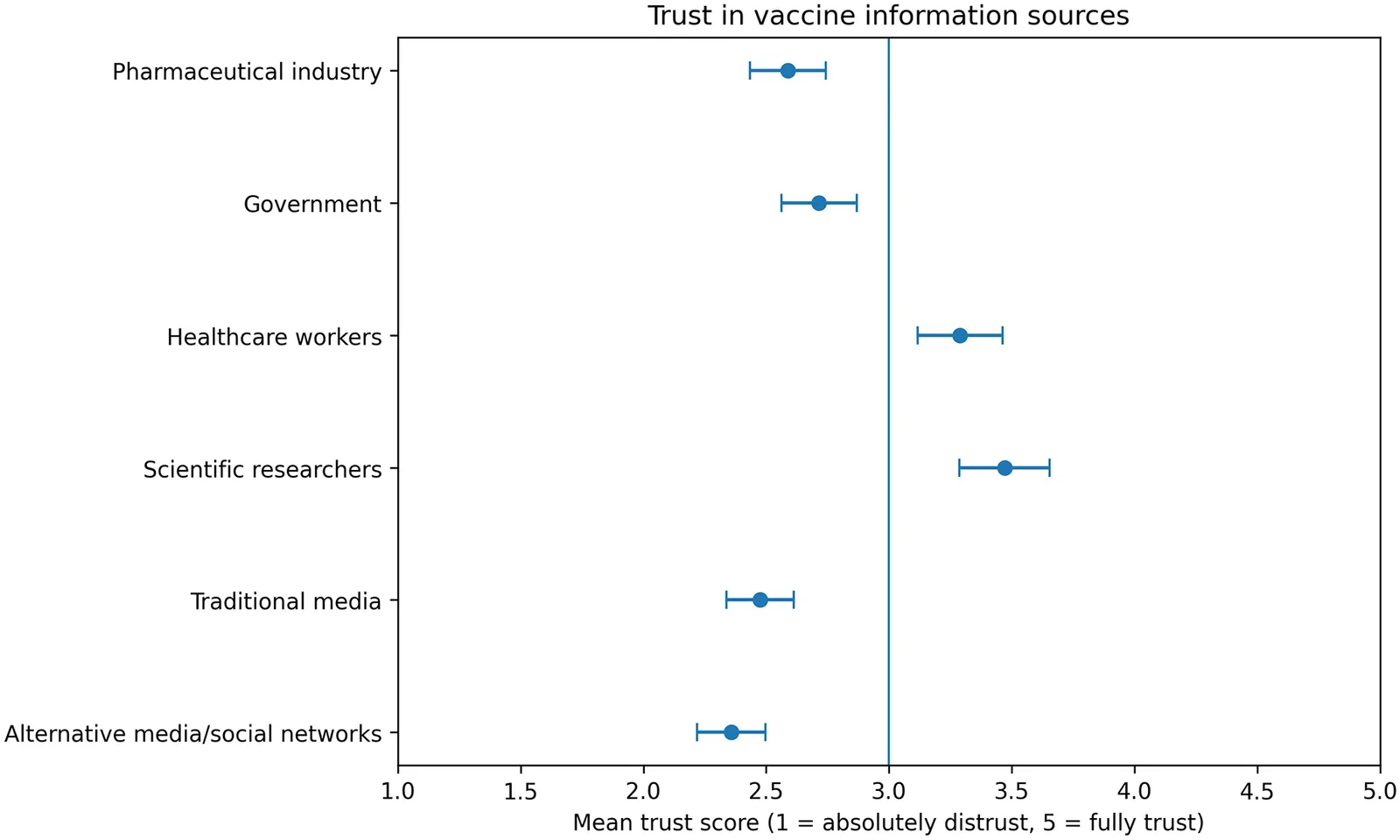
Mean trust in six vaccine information sources with 95% confidence intervals. Scores range from 1 (absolute distrust) to 5 (full trust); the vertical line marks neutrality.

### 3.3 Structure of HPV vaccine perceptions

The 16 attitude items were suitable for factor analysis (KMO = 0.749; Bartlett's test of sphericity, *χ*^2^ = 2846.2, df = 120, *p* < 0.001). Parallel analysis supported a two-factor solution (Supplementary Figure S1). The first factor primarily reflected perceived effectiveness together with social endorsement and service facilitation, whereas the second reflected safety- and age-related concern. Because the first factor was conceptually heterogeneous, the regression outcomes were constructed from coherent item subsets supported by both psychometric performance and content rather than from complete factor scores. Full oblimin-rotated factor loadings and uniqueness estimates are presented in Supplementary Table S2. The direct item stating that HPV vaccines were unsafe showed weak, directionally inconsistent loading and high uniqueness; its standalone psychometric performance is reported in Supplementary Table S7.

The preliminary five-item safety concern measure had a mean of 2.34 ± 0.97, Cronbach's *α* = 0.868, and McDonald's *ω* = 0.901. The preliminary three-item perceived effectiveness measure had a mean of 3.20 ± 1.24, *α* = 0.922, and *ω* = 0.948. Descriptive and reliability statistics for the analytical measures are summarized in Table 2. The two scores were only weakly correlated (Spearman's *ρ* = 0.129, *p* = 0.055), indicating that perceived effectiveness and safety concern were not simple opposites in this sample.

**Table 2:** Descriptive and reliability statistics for the analytical measures.

Measure	Items	Range	Mean	SD	Median (Q1-Q3)	*α*	*ω*
HPV knowledge	12	0–12	4.95	3.84	5.00 (1.00–8.00)	0.886	
Overall trust	6	1–5	2.82	0.94	3.00 (2.00–3.33)	0.875	0.903
Safety concern	5	1–5	2.34	0.97	2.20 (1.60–3.00)	0.868	0.901
Perceived effectiveness	3	1–5	3.20	1.24	3.00 (2.00–4.00)	0.922	0.948

Knowledge was inversely associated with safety concern (Spearman's *ρ* = −0.284, *p* < 0.001) and positively associated with perceived effectiveness (*ρ* = 0.393, *p* < 0.001). Knowledge was also positively associated with professional/scientific trust (*ρ* = 0.342, *p* < 0.001), and perceived effectiveness had its strongest trust association with professional/scientific sources (*ρ* = 0.549, *p* < 0.001).

### 3.4 Factors associated with HPV vaccine safety concern

The extended safety concern model accounted for 44.6% of outcome variance (adjusted R^2^ = 0.403). Greater COVID-19 controversy spillover was associated with higher safety concern (B = 0.190, 95% CI 0.043−0.337, *p* = 0.012), as was distrust attributed to the earlier Kazakhstan HPV vaccination programme (B = 0.197, 95% CI 0.047−0.347, *p* = 0.010) and preference for natural immunity (B = 0.137, 95% CI 0.040−0.234, *p* = 0.006). Healthcare workers had modestly lower adjusted concern than other participants (B = −0.225, 95% CI −0.449 to −0.001, *p* = 0.049). The adjusted association between knowledge and safety concern was attenuated after contextual belief items were included (B = −0.023, *p* = 0.269). Full model estimates are reported in Supplementary Table S3. Adjusted associations with HPV vaccine safety concern are shown in Figure 3.

**Figure 3:**
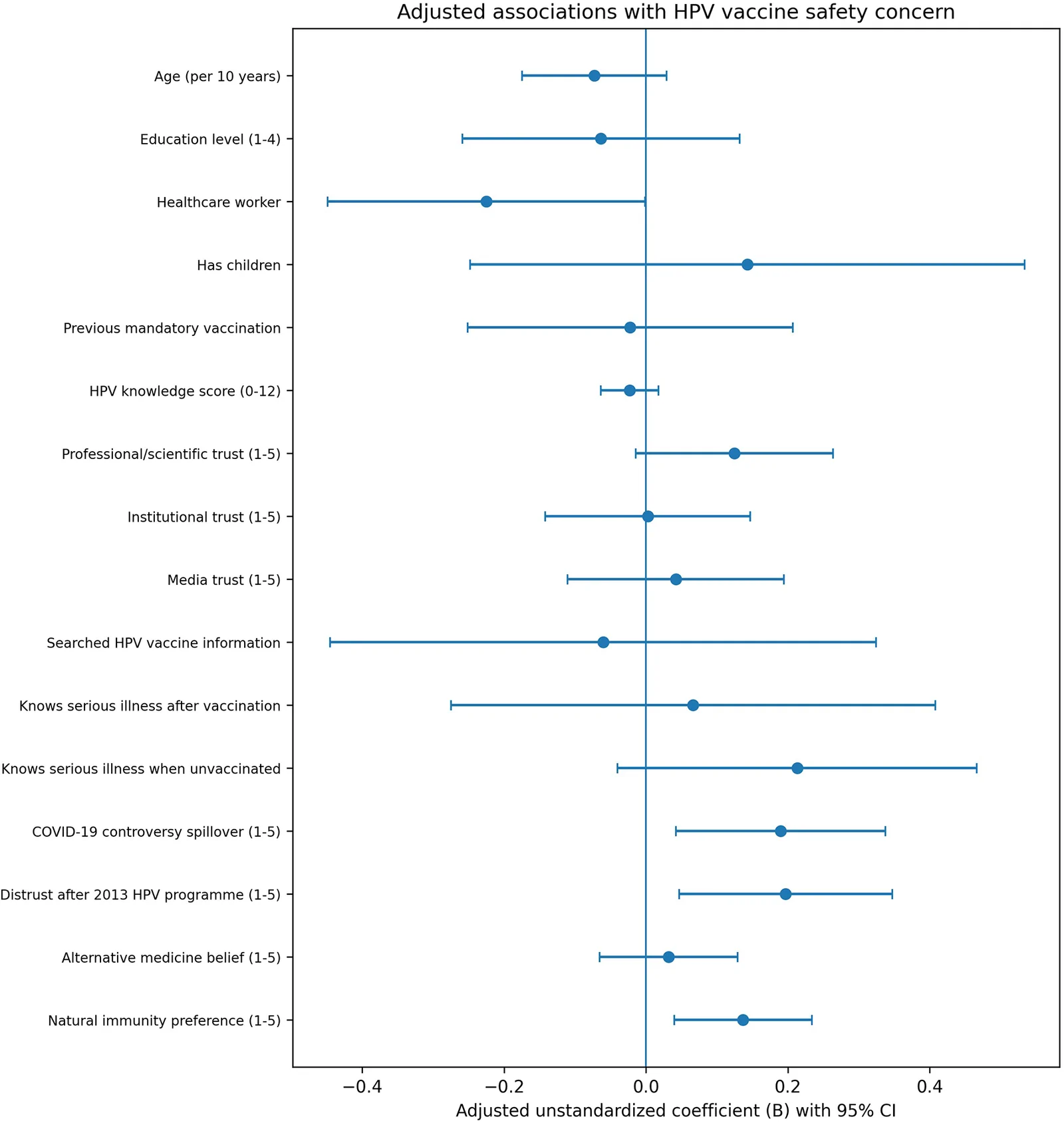
Adjusted associations with the five-item HPV vaccine safety concern score. Points are unstandardized coefficients and lines are 95% confidence intervals.

### 3.5 Factors associated with perceived effectiveness

The perceived effectiveness model accounted for 37.5% of variance (adjusted R^2^ = 0.339). Each additional correct knowledge answer was associated with a 0.061-point higher effectiveness score (95% CI 0.010−0.111, *p* = 0.019). Professional/scientific trust was the strongest independent correlate: each one-point increase was associated with a 0.297-point higher effectiveness score (95% CI 0.114−0.480, *p* = 0.001). Other sociodemographic, institutional-trust, media-trust, and anecdotal-experience variables were not independently associated with effectiveness. Full model estimates are reported in Supplementary Table S4. Adjusted associations with perceived HPV vaccine effectiveness are shown in Figure 4.

**Figure 4:**
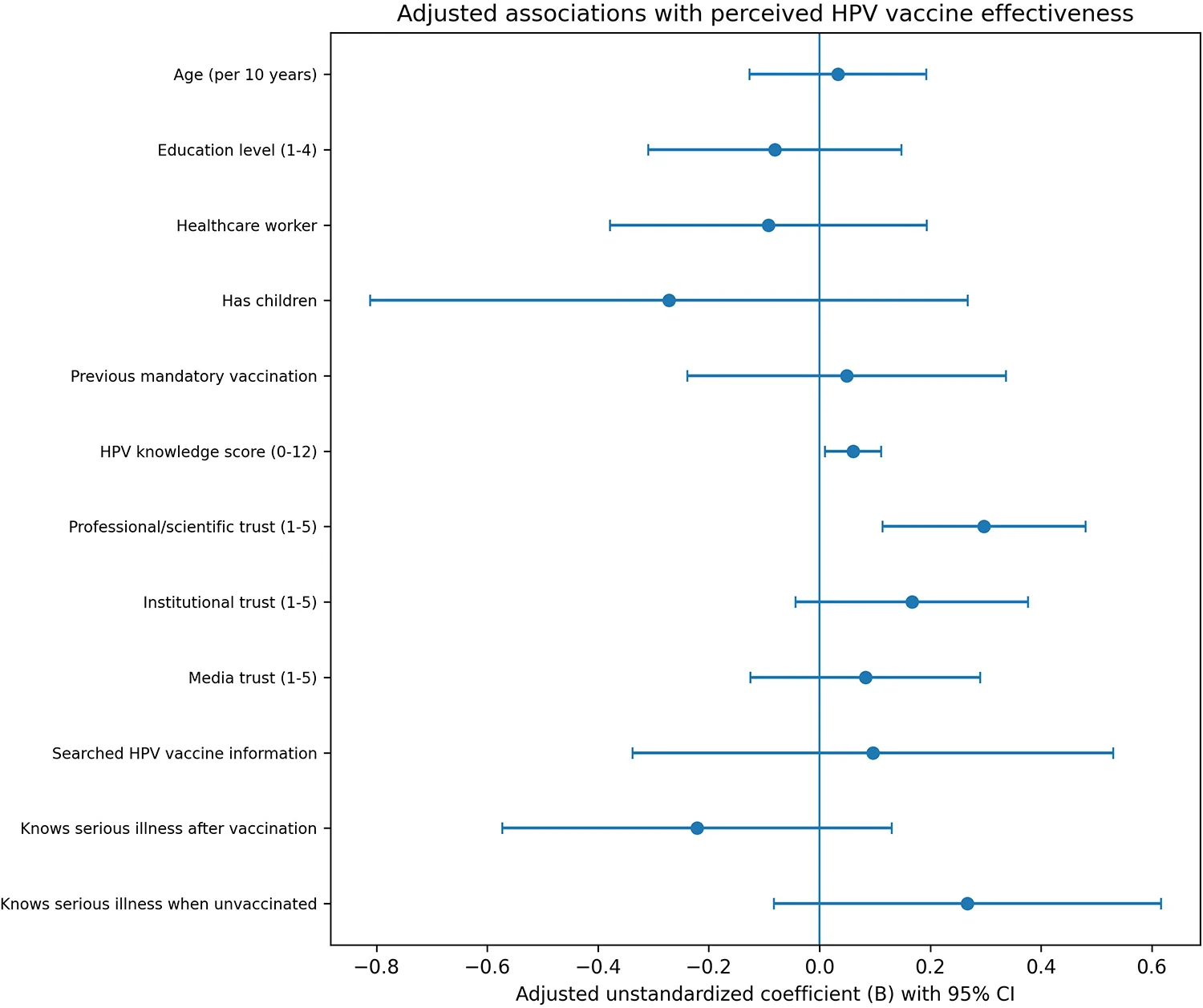
Adjusted associations with the three-item perceived HPV vaccine effectiveness score. Points are unstandardized coefficients and lines are 95% confidence intervals.

### 3.6 Secondary and sensitivity analyses

In a secondary exploratory analysis, 49 participants (22.2%) reported refusal or intention to refuse HPV vaccination for a daughter. Having children was associated with higher odds of this response (aOR = 3.71, 95% CI 1.40−9.81, *p* = 0.008), and knowing someone believed to have become seriously ill after vaccination showed the strongest association (aOR = 7.42, 95% CI 2.60−21.16, *p* < 0.001). Because the item combined actual refusal among women with a daughter and prospective refusal intention among women without a daughter, these estimates should not be interpreted as the prevalence or determinants of verified historical HPV vaccine refusal. Full model estimates and apparent performance measures are presented in Supplementary Table S5.

Sensitivity analyses repeated the primary models among non-healthcare workers, participants with children, and participants who had heard of the HPV vaccine. The principal pattern was broadly maintained: natural-immunity preference remained associated with safety concern in key subgroups, and professional/scientific trust remained associated with perceived effectiveness. Subgroup estimates are provided in Supplementary Table S6.

## 4 Discussion

### 4.1 Principal findings

Four principal findings emerged. First, the HPV vaccine perception items indicated distinct and only weakly related safety- and effectiveness-related dimensions, from which preliminary analytical composites were derived. Second, higher knowledge was associated with lower safety concern in bivariate analysis and with stronger perceived effectiveness after adjustment. Third, safety concern was most strongly associated with spillover from COVID-19 vaccine controversies, distrust related to Kazakhstan's earlier HPV vaccination programme, and preference for natural immunity. Finally, anecdotal exposure to a serious post-vaccination illness narrative was strongly associated with the exploratory daughter-directed refusal or refusal-intention response.

### 4.2 Vaccine perceptions are multidimensional

The weak correlation between safety concern and perceived effectiveness is substantively important. A woman may believe that HPV vaccination prevents cancer while simultaneously fearing long-term harms or regarding the recommended age as too young. Combining these responses into one total score can obscure actionable differences and produce misleading associations. Contemporary models of vaccine decision-making distinguish confidence in safety and effectiveness from convenience, collective responsibility, risk appraisal, social norms, and institutional trust (8, 18). In the present analysis, the broader first factor also contained social-endorsement and service-facilitation items; accordingly, the safety concern and perceived effectiveness composites were constructed as conceptually coherent subsets rather than treated as complete factor scores. Their use should therefore be understood as exploratory and study-specific pending independent validation.

The poor performance of the direct “HPV vaccines are unsafe” item deserves particular attention. Its response distribution and inverse correlations were inconsistent with the other negatively framed items. The item wording and response direction were those of the validated bilingual questionnaire, and verification against the scoring framework did not identify a coding or reverse-scoring discrepancy. Possible explanations therefore include item-specific interpretation, response-process effects, or a genuine difference between a global safety judgment and more specific concerns about long-term effects and novelty. Cognitive interviewing and independent psychometric testing would be useful before incorporating this item into a future composite measure.

### 4.3 Knowledge, trust, and perceived effectiveness

Women with greater HPV knowledge had fewer safety concerns and stronger perceptions of effectiveness. However, knowledge alone accounted for only part of the variation. Professional and scientific trust was independently associated with perceived effectiveness and had the largest standardized effect in that model. This aligns with systematic evidence showing that healthcare-provider recommendation and communication are among the strongest correlates of HPV vaccine uptake (9, 11). It also agrees with Kazakhstan studies identifying healthcare professionals as central to communication while documenting gaps in provider knowledge and recommendation practice (15, 16).

The practical implication is not simply to increase the quantity of information. Communication should connect factual knowledge to a clear cancer-prevention recommendation delivered by trained clinicians. Provider-facing interventions should include concise evidence on vaccine safety, effectiveness, age rationale, continued cervical screening, and response strategies for common misinformation. Systematic reviews support strong and presumptive recommendations followed by respectful discussion of concerns, and a recent meta-analysis of randomized studies found that clinician communication training improved HPV vaccination uptake, with larger effects for presumptive communication strategies (11, 19, 27).

### 4.4 Historical and contemporary narratives of safety

The associations of safety concern with the earlier Kazakhstan programme and with COVID-19 controversy spillover suggest that HPV vaccination is interpreted through a broader history of vaccine confidence. Kazakhstan's earlier pilot was affected by low uptake, parental refusal, negative media coverage, and persistent safety narratives, while healthcare professionals also reported communication barriers (15, 16, 21). The renewed programme therefore does not begin from a neutral baseline. Transparent acknowledgement of that history, explanation of adverse-event assessment, and visible safety monitoring may be more credible than communication that simply repeats that vaccination is safe.

The COVID-19 pandemic further altered vaccine discourse. The current data suggest cross-vaccine spillover: controversy surrounding one vaccine platform can reduce confidence in unrelated routine recommendations. International qualitative work similarly shows that COVID-19 became a dominant topic in later HPV vaccine conversations, while social-media research documents recurring narratives around injury, necessity, conspiracy, and mistrust (12, 20). Communication campaigns should therefore monitor the information environment and address transferable vaccine narratives rather than treating HPV misinformation as an isolated topic.

### 4.5 Natural immunity and anecdotal experience

Preference for natural immunity remained independently associated with greater safety concern. For HPV, the natural-infection framing is particularly problematic because infection is common, may persist without symptoms, cannot be relied upon to generate protective immunity against future disease, and can lead to cancer. Communication should contrast the predictable immune protection of vaccination with the uncertain and potentially harmful consequences of natural infection without using moralizing or dismissive language.

The strong association between knowing someone believed to have become seriously ill after vaccination and the daughter-directed refusal or refusal-intention response illustrates the potential influence of personal narratives. This variable does not establish that the illness was vaccine-caused; it measures a socially transmitted attribution. Likewise, the daughter outcome combines prior behaviour and prospective intention and should not be interpreted as verified HPV vaccine uptake. Statistical safety data often compete poorly with vivid anecdotes. Clinicians and public health communicators should acknowledge the experience, distinguish temporal association from causation, explain how adverse events are evaluated, and provide an accessible route for reporting and follow-up.

### 4.6 Implications for the Kazakhstan vaccination programme

Four programme-level implications follow. First, information materials should separately address effectiveness and safety rather than assuming that belief in benefit removes concern. Second, healthcare workers require standardized communication training and rapid access to evidence-based answers because professional-scientific trust is a key asset and communication training can improve HPV vaccine uptake (11, 19, 27). Third, the history of the earlier national HPV vaccination programme should be addressed directly through transparent safety surveillance and communication. Fourth, intervention evaluation should measure actual first-dose and series-completion outcomes rather than attitude or intention measures alone. The present survey can inform message design, but it cannot estimate current vaccination coverage.

### 4.7 Strengths and limitations

The study has several strengths. It analyses a contemporary sample after national programme reintroduction, includes detailed knowledge, trust, information, and contextual items, and focuses on an underrepresented Central Asian setting. The analysis used transparent scoring procedures, evaluated dimensional structure before constructing analytical outcomes, reported reliability beyond Cronbach's alpha, applied robust multivariable models, and included sensitivity analyses. The use of a validated bilingual questionnaire and the inclusion of representative item wording in the supplementary material further support transparency and reproducibility.

The study also has important limitations. Participants were recruited through open convenience sampling at one primary healthcare centre in Astana, and a conventional response rate could not be calculated. Women attending primary care may differ from the broader female population in healthcare engagement, access to information, and trust in healthcare professionals, and the sample also included a high proportion of healthcare workers. The cross-sectional design cannot establish temporal or causal direction. All measures were self-reported and may be affected by social desirability or recall. The daughter-directed refusal item intentionally applied to all respondents by combining actual refusal with prospective intention for a future daughter; it therefore cannot estimate verified historical refusal, and information on the presence, age, vaccination eligibility, or actual HPV vaccination status of daughters was unavailable. The factor structure and preliminary study-derived composites were developed and evaluated in the same modest sample, so confirmatory factor analysis and external validation are required. The direct unsafe item showed atypical psychometric performance and requires further response-process evaluation. The study did not measure the frequency or intensity of social-media exposure or the quality of provider communication. Finally, the apparent discrimination of the logistic model may be optimistic because internal and external validation were not performed.

## 5 Conclusion

Among women attending a single primary care centre in Astana, HPV vaccine safety concern and perceived effectiveness emerged as distinct analytical dimensions. Higher knowledge and professional-scientific trust were associated mainly with perceived effectiveness, whereas safety concern was more strongly associated with COVID-19 controversy spillover, distrust rooted in Kazakhstan's earlier HPV vaccination programme, and preference for natural immunity. In the exploratory daughter-directed analysis, personal narratives of serious illness after vaccination were strongly associated with refusal or intention to refuse vaccination for a daughter. These findings support communication strategies combining clear cancer-prevention recommendations, transparent safety dialogue, clinician training, and direct engagement with historical and contemporary vaccine narratives.

## Data Availability

The raw data supporting the conclusions of this article will be made available by the authors, without undue reservation.
