# Supplementary material for: Knowledge, trust, and multidimensional perceptions of human papillomavirus vaccination among women attending a single primary care centre in Astana, Kazakhstan: a cross-sectional study
Source: Front Glob Womens Health. 2026 Oct 8;7:1960252. doi: 10.3389/fgwh.2026.1960252 (PMC13687039; doi:10.3389/fgwh.2026.1960252)
Supplement: Supplementary file 1 [file Table1.docx]

**Supplementary Material**

# Supplementary methods

## Scoring framework

All five-category response items were scored according to the semantic order of the questionnaire response options before composite scores were calculated. Agreement items ranged from 1 (fully disagree) to 5 (fully agree), and trust items ranged from 1 (absolute distrust) to 5 (full trust). The numeric values stored in the source dataset were treated as category identifiers and mapped to these ordered analytical scores. Knowledge items were scored 1 for a correct response and 0 for an incorrect or 'don't know' response. Composite scores were calculated as the mean or sum of the specified items, as detailed in Supplementary Table S1.

## Psychometric analysis

The 16 HPV vaccine perception items were evaluated using a polychoric correlation matrix. Sampling adequacy was assessed using the Kaiser-Meyer-Olkin statistic and Bartlett's test of sphericity. Factor retention was guided by parallel analysis with 1,000 random permutations, followed by principal-axis exploratory factor analysis with oblimin rotation. The analysis was exploratory and was used to characterize dimensional structure rather than to confirm prespecified latent scales. Final analytical composites were constructed from conceptually coherent item subsets supported by factor loadings, cross-loadings, uniqueness, item-total behaviour, and content. Full factor loadings are reported in Supplementary Table S2 and the parallel analysis is shown in Supplementary Figure S1.

## Sensitivity analyses

The primary multivariable models were repeated in three analytical subgroups: non-healthcare workers, participants with children, and participants who had heard of the HPV vaccine. The subgroup estimates are summarized in Supplementary Table S6.

*Supplementary Table S1. Recoding and construction of analytical scores*

| **Component / item group** | **Stored code or included items** | **Response category or calculation** | **Analytical score / range** | **Direction** |
| --- | --- | --- | --- | --- |
| **A. Stored-code recoding** | | | | |
| Contextual agreement items | 2 | Fully disagree | 1 | Higher = greater agreement |
| Contextual agreement items | 1 | Somewhat disagree | 2 |  |
| Contextual agreement items | 0 | Neutral | 3 |  |
| Contextual agreement items | 3 | Somewhat agree | 4 |  |
| Contextual agreement items | 4 | Fully agree | 5 |  |
| Trust items | 4 | Absolutely distrust | 1 | Higher = greater trust |
| Trust items | 3 | Partly distrust | 2 |  |
| Trust items | 0 | Neutral | 3 |  |
| Trust items | 1 | Somewhat trust | 4 |  |
| Trust items | 2 | Fully trust | 5 |  |
| HPV attitude agreement items | 4 | Fully disagree | 1 | Higher = greater agreement |
| HPV attitude agreement items | 3 | Somewhat disagree | 2 |  |
| HPV attitude agreement items | 0 | Neutral | 3 |  |
| HPV attitude agreement items | 1 | Somewhat agree | 4 |  |
| HPV attitude agreement items | 2 | Fully agree | 5 |  |
| **B. Composite-score construction** | | | | |
| HPV knowledge score | 12 true/false/don't-know items | Sum of correct responses | 0-12 | Higher = greater knowledge |
| Professional/scientific trust | Healthcare professionals; scientific researchers | Mean of two 1-5 trust items | 1-5 | Higher = greater trust |
| Institutional trust | Pharmaceutical industry; government | Mean of two 1-5 trust items | 1-5 | Higher = greater trust |
| Media trust | Traditional media; alternative media/social networks | Mean of two 1-5 trust items | 1-5 | Higher = greater trust |
| Overall trust | All six trust items | Mean of six 1-5 trust items | 1-5 | Higher = greater trust |
| Safety concern | Long-term health problems; insufficient study; too new to judge safety; daughter too young; not mandatory because too dangerous | Mean of five 1-5 agreement items | 1-5 | Higher = greater concern |
| Perceived effectiveness | Prevents HPV infection; prevents genital warts; prevents HPV-related cancer | Mean of three 1-5 agreement items | 1-5 | Higher = greater perceived effectiveness |

*Supplementary Table S2. Full oblimin-rotated exploratory factor pattern matrix*

| **Item** | **Abbreviated item wording** | **Factor 1** | **Factor 2** | **Uniqueness** | **Analytical use** |
| --- | --- | --- | --- | --- | --- |
| BI | May cause long-term health problems | -0.071 | -0.754 | 0.452 | Safety concern |
| BJ | HPV vaccines are insufficiently studied | 0.205 | -0.714 | 0.378 | Safety concern |
| BK | HPV vaccines are unsafe | -0.003 | 0.254 | 0.935 | Standalone item |
| BL | Effectively prevents HPV infection | 0.954 | 0.095 | 0.125 | Perceived effectiveness |
| BM | Effectively prevents genital warts | 0.862 | -0.078 | 0.218 | Perceived effectiveness |
| BN | Vaccinating daughter helps protect against STIs | 0.116 | -0.416 | 0.790 | Not included in primary composite |
| BO | Effectively prevents HPV-related cancer | 0.979 | 0.112 | 0.082 | Perceived effectiveness |
| BP | Condom use prevents HPV transmission | 0.564 | -0.332 | 0.482 | Not included in primary composite |
| BQ | Pap testing can prevent cervical cancer | 0.483 | -0.335 | 0.577 | Not included in primary composite |
| BR | Most friends vaccinate their daughters | 0.593 | -0.255 | 0.511 | Not included in primary composite |
| BS | Healthcare workers consider vaccination a good idea | 0.392 | 0.439 | 0.737 | Not included in primary composite |
| BT | Too new to know whether it is safe and reliable | 0.165 | -0.790 | 0.286 | Safety concern |
| BU | Same-day availability would facilitate vaccination | 0.800 | 0.026 | 0.370 | Not included in primary composite |
| BV | If important, the vaccine would be mandatory | 0.672 | -0.259 | 0.397 | Not included in primary composite |
| BW | Daughter is too young for HPV vaccination | 0.046 | -0.755 | 0.411 | Safety concern |
| BX | Not mandatory because it is too dangerous | -0.166 | -0.902 | 0.231 | Safety concern |

*KMO = 0.749; Bartlett's test of sphericity χ² = 2846.2, df = 120, p < 0.001.*

*Supplementary Figure S1. Parallel analysis for the 16 HPV vaccine perception items*

*Supplementary Table S3. Full robust multivariable model for HPV vaccine safety concern*

| **Predictor** | **B** | **95% CI** | **Standardized β** | **p** |
| --- | --- | --- | --- | --- |
| Age (per 10 years) | -0.073 | -0.174 to 0.029 | -0.116 | 0.162 |
| Education level (1-4) | -0.063 | -0.259 to 0.132 | -0.045 | 0.525 |
| Healthcare worker | -0.225 | -0.449 to -0.001 | -0.114 | 0.049 |
| Has children | 0.143 | -0.248 to 0.534 | 0.073 | 0.474 |
| Previous mandatory vaccination | -0.022 | -0.252 to 0.207 | -0.011 | 0.849 |
| HPV knowledge score (0-12) | -0.023 | -0.064 to 0.018 | -0.091 | 0.269 |
| Professional/scientific trust (1-5) | 0.125 | -0.014 to 0.264 | 0.166 | 0.079 |
| Institutional trust (1-5) | 0.003 | -0.142 to 0.147 | 0.003 | 0.971 |
| Media trust (1-5) | 0.042 | -0.110 to 0.194 | 0.042 | 0.589 |
| Searched HPV vaccine information | -0.060 | -0.445 to 0.324 | -0.022 | 0.758 |
| Knows serious illness after vaccination | 0.067 | -0.275 to 0.408 | 0.024 | 0.703 |
| Knows serious illness when unvaccinated | 0.213 | -0.040 to 0.466 | 0.107 | 0.099 |
| COVID-19 controversy spillover (1-5) | 0.190 | 0.043 to 0.337 | 0.252 | 0.012 |
| Distrust after 2013 HPV programme (1-5) | 0.197 | 0.047 to 0.347 | 0.242 | 0.010 |
| Alternative medicine belief (1-5) | 0.032 | -0.065 to 0.129 | 0.042 | 0.519 |
| Natural immunity preference (1-5) | 0.137 | 0.040 to 0.234 | 0.207 | 0.006 |

*Model R² = 0.446; adjusted R² = 0.403. Higher outcome scores indicate greater safety concern.*

*Supplementary Table S4. Full robust multivariable model for perceived HPV vaccine effectiveness*

| **Predictor** | **B** | **95% CI** | **Standardized β** | **p** |
| --- | --- | --- | --- | --- |
| Age (per 10 years) | 0.033 | -0.127 to 0.193 | 0.042 | 0.684 |
| Education level (1-4) | -0.080 | -0.309 to 0.148 | -0.044 | 0.490 |
| Healthcare worker | -0.092 | -0.378 to 0.194 | -0.037 | 0.528 |
| Has children | -0.272 | -0.811 to 0.268 | -0.109 | 0.324 |
| Previous mandatory vaccination | 0.049 | -0.238 to 0.337 | 0.020 | 0.737 |
| HPV knowledge score (0-12) | 0.061 | 0.010 to 0.111 | 0.188 | 0.019 |
| Professional/scientific trust (1-5) | 0.297 | 0.114 to 0.480 | 0.311 | 0.001 |
| Institutional trust (1-5) | 0.167 | -0.043 to 0.377 | 0.144 | 0.119 |
| Media trust (1-5) | 0.083 | -0.124 to 0.290 | 0.065 | 0.433 |
| Searched HPV vaccine information | 0.096 | -0.337 to 0.530 | 0.028 | 0.663 |
| Knows serious illness after vaccination | -0.221 | -0.573 to 0.131 | -0.063 | 0.218 |
| Knows serious illness when unvaccinated | 0.267 | -0.082 to 0.617 | 0.105 | 0.134 |

*Model R² = 0.375; adjusted R² = 0.339. Higher outcome scores indicate greater perceived effectiveness.*

*Supplementary Table S5. Secondary logistic model for reported refusal or intention to refuse HPV vaccination for a daughter*

| **Predictor** | **Adjusted OR** | **95% CI** | **p** |
| --- | --- | --- | --- |
| Age (per 10 years) | 0.97 | 0.72 to 1.32 | 0.869 |
| Has children | 3.71 | 1.40 to 9.81 | 0.008 |
| HPV knowledge score (0-12) | 0.97 | 0.89 to 1.07 | 0.564 |
| Safety concern (1-5) | 1.45 | 0.89 to 2.37 | 0.136 |
| Perceived effectiveness (1-5) | 0.67 | 0.44 to 1.02 | 0.060 |
| Professional/scientific trust (1-5) | 0.88 | 0.66 to 1.18 | 0.404 |
| Knows serious illness after vaccination | 7.42 | 2.60 to 21.16 | <0.001 |
| Religious vaccine refusal | 3.06 | 0.46 to 20.46 | 0.249 |

*Outcome events = 49/221; apparent AUC = 0.839; Brier score = 0.121. The item applied to all respondents: women with a daughter answered with reference to actual refusal or a decision not to vaccinate, whereas women without a daughter were instructed to answer prospectively with reference to a future daughter. The outcome therefore represents a combined refusal/refusal-intention response rather than verified historical HPV vaccine refusal or observed uptake.*

*Supplementary Table S6. Sensitivity analyses of the primary multivariable models*

| **Subset** | **n** | **Safety model R²** | **Key safety-model estimates** | **Effectiveness model R²** | **Key effectiveness-model estimates** |
| --- | --- | --- | --- | --- | --- |
| All participants | 221 | 0.446 | COVID-19: 0.190 (0.043 to 0.337; p=0.012); Earlier programme distrust: 0.197 (0.047 to 0.347; p=0.010); Natural immunity: 0.137 (0.040 to 0.234; p=0.006) | 0.375 | Knowledge: 0.061 (0.010 to 0.111; p=0.019); Professional/scientific trust: 0.297 (0.114 to 0.480; p=0.001) |
| Non-healthcare workers | 131 | 0.465 | COVID-19: 0.164 (-0.067 to 0.396; p=0.163); Earlier programme distrust: 0.259 (-0.005 to 0.523; p=0.055); Natural immunity: 0.178 (0.038 to 0.317; p=0.013) | 0.441 | Knowledge: 0.082 (0.016 to 0.148; p=0.015); Professional/scientific trust: 0.255 (0.002 to 0.508; p=0.048) |
| Participants with children | 127 | 0.545 | COVID-19: 0.133 (-0.045 to 0.312; p=0.143); Earlier programme distrust: 0.246 (0.041 to 0.452; p=0.019); Natural immunity: 0.168 (0.034 to 0.303; p=0.014) | 0.418 | Knowledge: 0.048 (-0.022 to 0.119; p=0.178); Professional/scientific trust: 0.278 (0.052 to 0.505; p=0.016) |
| Participants aware/heard HPV vaccine | 210 | 0.470 | COVID-19: 0.213 (0.062 to 0.363; p=0.006); Earlier programme distrust: 0.184 (0.030 to 0.337; p=0.019); Natural immunity: 0.125 (0.027 to 0.224; p=0.012) | 0.401 | Knowledge: 0.045 (-0.004 to 0.093; p=0.074); Professional/scientific trust: 0.351 (0.169 to 0.533; p=0.000) |

*Entries are unstandardized B coefficients with 95% confidence intervals and p-values. Each subgroup model retained the same predictor specification as its corresponding primary model.*

*Supplementary Table S7. Psychometric performance of the direct item “HPV vaccines are unsafe”*

| **Metric** | **Estimate** | **Interpretation** |
| --- | --- | --- |
| Factor 2 loading | 0.254 | Weak relative to retained safety items |
| Uniqueness | 0.935 | Very high unexplained item variance |
| Spearman correlation with five-item safety concern | -0.182 (p = 0.007) | Directionally inconsistent with the retained safety items |
| Spearman correlation with perceived effectiveness | 0.025 (p = 0.711) | No evidence of association |
| Spearman correlation with HPV knowledge | 0.116 (p = 0.084) | No statistically significant association |
| Cronbach's α: five-item safety concern | 0.868 | Retained composite |
| Cronbach's α after adding the unsafe item | 0.752 | Internal consistency decreased |

*Supplementary Table S8. Representative questionnaire items and response formats used in the analysis*

| **Domain** | **Representative item(s)** | **Response format** | **Analytical use** |
| --- | --- | --- | --- |
| HPV knowledge | Examples: “HPV infections are very rare”; “A person can be infected with HPV for many years without knowing it”; “Girls who were vaccinated against HPV still need cervical cancer screening when they are older.” | True / False / Don't know | 12-item knowledge score; correct = 1, incorrect or don't know = 0 |
| Trust in information sources | “Please rate how much you trust the following sources to tell the truth about vaccines”: pharmaceutical industry; government; healthcare worker; scientific researchers; traditional media; alternative media/social networks. | 5-point scale: absolute distrust to full trust | Professional/scientific, institutional, media, and overall trust measures |
| Contextual vaccine beliefs | Examples: “Because of controversies surrounding COVID-19 vaccines, I trust HPV vaccination recommendations less”; “Because the HPV vaccination programme launched in Kazakhstan in 2013 was associated with complications in some cases, I trust the healthcare system less”; “I prefer my daughter … to develop immunity to HPV naturally rather than through vaccination.” | 5-point agreement scale | Predictors in the safety-concern model |
| Safety concern | Examples: “The HPV vaccine may cause long-term health problems”; “HPV vaccines have not been studied enough”; “The HPV vaccine is too new to know how safe and reliable it is”; “I think my daughter is too young for HPV vaccination”; “HPV vaccination is not mandatory because it is too dangerous.” | 5-point agreement scale | Five-item preliminary safety concern composite |
| Perceived effectiveness | “The HPV vaccine effectively prevents HPV infection”; “The HPV vaccine effectively prevents genital warts”; “The HPV vaccine effectively prevents HPV-related cancer.” | 5-point agreement scale | Three-item preliminary perceived effectiveness composite |
| Direct safety item | “HPV vaccines are unsafe.” | 5-point agreement scale | Standalone psychometric item; not included in safety composite |
| Daughter-directed refusal / intention | “I have refused a vaccine for my daughter (if you have a daughter, or in the future if you have a daughter), or decided not to have her vaccinated.” | Yes / No | Secondary exploratory logistic outcome; all participants answered |
| Anecdotal vaccine experience | “I know someone who became seriously ill after vaccination”; “I know someone who became seriously ill because they were not vaccinated.” | Yes / No | Regression predictors |
| Information seeking | “Have you ever searched for information about the HPV vaccine?” | Yes / No | Regression predictor and descriptive variable |

*Note: English renderings are provided to illustrate item content and response structure. The survey itself was administered using the validated Russian- and Kazakh-language questionnaire supplied by the instrument developers.*
